# Understanding the online landscape of cannabis discourse: a Twitter analysis

**DOI:** 10.3389/fpubh.2025.1416171

**Published:** 2025-07-17

**Authors:** Miguel Angel Alvarez-Mon, Carla Ojeda, Francisco Lara-Abelenda, Ángel Asunsolo del Barco, Oscar Fraile-Martínez, Cielo García-Montero, Sonia Fernández-Rojo, Javier Quintero, Miguel Angel Ortega, Melchor Alvarez-Mon, Fernando Mora

**Affiliations:** ^1^Department of Medicine and Medical Specialities, Faculty of Medicine and Health Sciences, University of Alcala, Alcala de Henares, Spain; ^2^Ramón y Cajal Institute of Sanitary Research (IRYCIS), Madrid, Spain; ^3^Department of Psychiatry and Mental Health, Hospital Universitario Infanta Leonor, Madrid, Spain; ^4^Departamento Teoria de la Señal y Comunicaciones y Sistemas Telemáticos y Computación, Escuela Tecnica Superior de Ingenieria de Telecomunicación, Universidad Rey Juan Carlos, Fuenlabrada, Spain; ^5^Department of Surgery, Medical and Social Sciences, Faculty of Medicine and Health Sciences, University of Alcalá, Alcala de Henares, Spain; ^6^Department of Epidemiology and Biostatistics, Graduate School of Public Health and Health Policy, University of New York, New York, NY, United States; ^7^Department of Legal and Psychiatry, Complutense University, Madrid, Spain; ^8^Service of Internal Medicine and Immune System Diseases-Rheumatology, University Hospital Príncipe de Asturias, (CIBEREHD), Alcalá de Henares, Spain

**Keywords:** cannabis, marihuana, hachis, Twitter, infodemiology

## Abstract

**Background and aims:**

Consultations and admissions for pathologies related to cannabis use are growing ininterrumpedly. The lack of public awareness of the risks can have a negative impact on our society, as well as on new policy proposals. In response, we set out to investigate social media posts about cannabis to better understand the online environment in this regard.

**Design, setting, and measures:**

The study will analyze a dataset of tweets posted between 2018 and 2022, written in Spanish, that include the keyword cannabis, marihuana, or hachis. A total of 68,673 tweets were included in our study. A subset of 500 posts for each keyword was manually analyzed by a researcher to establish a codebook. Subsequently, Machine Learning techniques were employed to analyze the remaining 67,173 comments using the established codebook. Finally, 32,646 of the remaining tweets were excluded as they contained information unrelated to the objectives of this study.

**Findings:**

Our research reveals a pronounced Twitter user engagement with cannabis, primarily centered on its regulatory and health dimensions. In more detail, 73.2% of the analyzed tweets were in favor and only 3.5% of the population expressed against its regulation, whereas only 20.4% of the tweets discussed the negative effects of cannabis on physical or mental health. Additionally, 30.1% of the tweets are in favor of the therapeutic use of cannabis, while 69.9% of tweets manifest neutral or against therapeutic use. Our findings also show significant differences on these topics depending on the user type and between consumers versus non-consumers.

**Conclusion:**

This analysis of tweets about cannabis provides information on experiences and opinions related to its use. Therefore, the perspectives of Twitter users constitute valuable input that can help improve physicians’ knowledge about cannabis and their communication with patients about its dangerousness.

## 1 Introduction

Cannabis, also known by the names of hashish and marijuana is the most commonly consumed illegal drug in the world (1). According to the World Health Organisation (WHO), about 147 million people (2.5% of the world population) consume cannabis annually, compared with 0.2% consuming cocaine and 0.2% consuming opiates (2). The most notable growth in cannabis abuse since the 1960s has been in developed countries, although developing countries are also increasing cannabis consumption (3, 4). According to the National Drug Plan of the Spanish government, 28.6% of the population between the ages of 14 and 18 have tried cannabis at least once in their lifetime and 22.2% have tried it in the last 12 months (5). In the population aged 15–64, cannabis is the most commonly consumed illegal drug in Spain and almost 41% of the surveyed have tried it at least once in their lifetime (6). Its consumption is more frequent among males and significantly decreases as age increases. Cannabis consumption rates in Spain have shown an increasing trend since 2013. On a European level, Spain ranks third in cannabis consumption, following France and Denmark (7, 8). Outside of Europe, other developed countries like the USA have also seen an increase in the number of consumers (9), while Hispanic American countries like Uruguay and Chile have also observed an increased consumption of cannabis over time, peaking among those aged 20–24 and increasing across all age groups, with period effects indicating notably higher prevalence in recent years, especially for women (10). Interestingly, other works have shown differences in Spanish-speaking people depending on their residence. A survey of 549 Spanish-speakers individuals, including 294 residing in the USA show that despite recreational use of cannabis was the most common topic, those living in the USA were more likely to consume daily cannabis smoked or vaporized, also reporting a similar interest with recreational and medical use pattern (11). Therefore, based on these data is of great importance to deepen on the perception of cannabis in Spanish-speaking people, considering specific differences across topics and regions.

Currently, there is a great controversy surrounding the effects and potential applications of the *Cannabis sativa* plant. On one hand, empirical evidence shows the multiple harms of its acute and chronic consumption when used recreationally, mainly through inhalation (12, 13). On the other hand, the approval of medical cannabis uses for certain conditions in an increasing number of places around the world has led to reforms in the regulatory laws concerning this substance, which in turn has had an impact on its consumption and perceived risk by the general population. In this regard, despite a considerable amount of studies examining cannabis consumption in all its various forms, often these research findings are not adequately synthesized, translated, or communicated to policymakers, healthcare providers, state health officials, and, in general, the entire population (14). In this context, not only cannabis consumption but also legalization issues represents a global subject of debate.

Multiple studies have been conducted on different social media like Twitter in order to understand the public opinion of a particular social concern, as it can be the uses, experiences and opinion around cannabis consumption (15–17). This platform has some particular advantages in comparison to surveys and other study designs, as it can be the perceived safety and facility of users to tweet about honest experiences without feeling judged or anonymity, observe the interactions between users, the accessibility and broad access to information, as well as the possibility to offer peer/social/emotional support, public health monitoring, and potential to influence health policy (18, 19). In this study, we aimed to investigate the public opinion regarding cannabis and its consumption in the context of the Spanish government considering its legalization. We set the following objectives: (1) Determine the main topic of Twitter publications, their generated interest, and their scientific adequacy; (2) understand the users’ perception of the health risks associated with cannabis consumption; (3) characterize the user types that are more supportive or against the legalization, considering therapeutic or recreational use; (4) considering geolocalization and cultural data to understand regional differences in the analyzed tweets.

## 2 Methods

### 2.1 Search and collection of tweets

This analysis focused on tweets related to cannabis posted on the social media platform Twitter. We included tweets that met the following criteria: (a) Public tweets; (b) Containing readable text in Spanish; (c) Using any of the keywords “cannabis,” “marihuana,” or “hachis” anywhere in the tweet; (d) Having received at least 10 retweets; (e) Published between January 1, 2018, and April 30, 2022.

We used the Tweet Binder tool to collect the tweets, which has been widely used in previous research and provides access to 100% of public tweets (20, 21). In addition to the tweet text, this tool provides the count of retweets and likes for each tweet, as well as the date of publication, a link to the tweet for contextual viewing, and user description. The number of retweets and likes received by each tweet was used as an indicator of the interest generated among users for the corresponding content (22–24).

The search resulted in a total of 247,156 tweets collected, out of which 178,483 were excluded as they were written in a language other than Spanish or contained too little text. Out of the remaining 68,673 tweets, a researcher analyzed 500 posts for each keyword (500 cannabis tweets, 500 marihuana tweets, and 500 hachís tweets) and established a codebook to analyze the remaining 67,173 comments using Machine Learning. Finally, 32,646 of the remaining tweets were excluded as they contained information unrelated to the objectives of this study or were written in a way that their meaning was uncertain.

### 2.2 Identification of thematic categories and creation of a codebook

The authors employed an inductive-deductive mixed approach to develop a codebook for classifying the content of the tweets based on key thematic categories. Deductively, they used categories from previous research that have also analyzed content posted on social media (25, 26). Inductively, they explored an initial subset of 1,500 tweets (from a small manually classified subset) to identify possible new themes and refine the codebook. Two researchers coded these 1,500 tweets, discussing any discrepancies with the research team and reaching a final consensus on coding. Once the final codebook was agreed upon, the machine coded the remaining 67,173 tweets, of which 34,528 could be analyzed, as the rest were unclassifiable.

The tweets were classified as classifiable or unclassifiable. A tweet was considered unclassifiable if its content was purely political, if the information was irrelevant to the objectives of the current work, or if it was a joke, uncertain or insufficient content. Among the tweets considered classifiable, it was determined whether the content was medical or non-medical, with these categories being mutually exclusive. In turn, the medical tweets were classified according to the clinical area of interest mentioned or discussed in the tweet text: (1) Health risks; (2) Discussion of therapeutic or medicinal use; (3) Content on preventive measures; (4) Sentiment regarding consumption; (5) Type of consumption. In the medical content tweets, it was evaluated whether they addressed the legalization debate or were related to recreational use of cannabis. Lastly, the users were classified into three categories: (1) General Twitter users and healthcare professionals (psychiatrists or doctors from other specialties, psychologists, nurses, clinical researchers, etc.); (2) Media and governmental (health institution) and non-governmental organizations (pro-cannabis associations); and (3) Public figures (politicians). In cases where tweets with nearly identical content were found, they were classified in the same way as the first tweet encountered. The classification criteria and examples of tweets are shown in Table 1.

**Table 1:** Examples of tweets.

*Classifiable tweets that are useful for our study and are analyzable* They may contain medical or non-medical information.	We open registrations for the Medicinal Cannabis Personal Cultivation Course for the month of May, aiming to promote self-cultivation as a means of access to cannabis. More information at https://t.co/i4w3gkITzO https://t.co/el709FfPaz Europe publishes its first manual for politicians interested in legalizing cannabis. https://t.co/m2jIemDokx It’s frustrating and disheartening to know about the persecution of cannabis patients and witness how drug trafficking grows day by day in our communities. I wonder why they do not put a stop to this? Prosecutors, these traffickers are criminals and attract delinquents!! https://t.co/ZsFVD0MhMz
*Health risks/harm* In favor of or against the existence of negative consequences on health.	How many people died from cannabis intoxication? How many individuals lost their lives in the fight against the “dreaded weed”? Shouldn’t we invest in healthcare instead? It is challenging to have conclusive evidence, but the available longitudinal information points to a significant relationship between cannabis consumption and the risk of developing a psychotic disorder. https://t.co/2YJEeeO9zM On #WorldPainDay, we remember that 17% of Spaniards suffer from chronic pain, and over two million people take anxiolytics daily. The regulation of cannabis and the proposed Mental Health Law will help alleviate the pain of millions of Spaniards. https://t.co/nXN5mMc5xC
*Legalization* Whether one is in favor or against the regulation of the sale and consumption of cannabis by the state.	Toward marijuana legalization 👇👇👇👇 https://t.co/DEhNAlV5Ud Scientific entrepreneurs and even two political parties join the cause of marijuana legalization. @EqInvestigacion https://t.co/qMhlpTht84 Hidden in the engine, they were carrying $2 million worth of marijuana; when divided, it was enough to supply 130 thousand people. Congratulations to @gendarmeria and @PFAOficial for apprehending the 2 drug traffickers in #Catamarca and preventing the drugs from reaching the neighborhoods. #ArgentinaSinNarcotráfico https://t.co/YQTVz6XcNR
*Therapeutic use* Support for cannabis as medicine.	The benefits of using *Cannabis sativa* for medicinal purposes are already well proven. Washington DC grants access to medical cannabis to all individuals over the age of 65. The city council has approved an initiative that allows them to self-certify as medical users and access dispensaries. → https://t.co/8o7T6YNxZ8 https://t.co/sAkdvWgnkR With great joy, we begin the harvest of 35 hectares of medicinal cannabis in public production. It will be the largest in Latin America, developed under the highest standards of good agricultural and post-harvest collection practices, and GMP. Happy harvest start, @CannavaSE! https://t.co/EMjFr2593v
*Opinion regarding cannabis consumption* Whether it is seen as something beneficial or detrimental at an individual and population level in different aspects: economic, social, etc.	@latercera My daughter is a medical user, and we went through a rough time with the police raid, but cannabis gave me another daughter who now has seizures only twice a month, compared to the 25 or more she used to have. @MamaCultiva Half of the young people with psychosis and schizophrenia are consumers of cannabis. https://t.co/icNxCi8XJx via @elcorreo_com Not a joke, friends. I think @VicenteFoxQue is losing his sanity! Symptoms of hallucination and paranoia are consequences of abusing opioids or cannabis!! #AmloElMejorPresidenteDelMundo #AMLOSíMeRepresenta https://t.co/YHW9rzc3wi
*Type of consumption* Where the person clearly expresses that they consume cannabis or, on the contrary, does not talk about it or denies it.	It seems like you are expressing frustration about people criticizing or objecting to others posting content related to smoking cannabis. You also mentioned that since you started using cannabis oil, your skin has improved significantly. However, you feel bothered when others post pictures of their future ex-partner with affectionate captions, as it may lead to heartbreak. For you, cannabis has been a source of healing.

### 2.3 Ethical considerations

This study has been conducted in accordance with the ethical research principles outlined in the Declaration of Helsinki (seventh revision, 2013) and has been approved by the ethics committee of the Complutense University. In any case, it did not directly involve human subjects nor included any interventions. Furthermore, we have taken care not to directly disclose any usernames in this study and have avoided citing information that could identify specific individuals.

### 2.4 Machine learning classifier

Recent technological advances have led to the emergence of artificial intelligence (AI), which can process and analyze data (27). Machine Learning (ML) is a branch of AI that focuses on extracting knowledge from data using computational models. Deep Learning (DL), a subset of ML, employs neural networks inspired by the human brain to process information (28). Neural networks have various applications, including weather prediction (29) or object recognition (30). Besides, Natural Language Processing (NLP) extensively utilizes neural networks to analyze text, recreate conversations, or and extract key ideas (31). In this project, a pretrained network called BERTWEET, trained on 850 million English tweets, was used to classify cannabis-related tweets (32).

Before implementing BERTWEET, the database underwent preprocessing. Non-English tweets were translated to English using Google Translator, and the tweets were normalized by removing special characters, splitting negative contractions, and eliminating repetitions. Since BERTWEET was not initially trained for the specific categories, fine-tuning was performed. The manually classified tweets were randomly divided into an 70% training subset and a 30% testing subset. The training subset was used to fine-tune the network, and the testing subset validated its performance. Additionally, the training set contains imbalanced categories, with varying counts across different options. To solve that, we employed the easy data augmentation (EDA) pipeline (33) to generate additional tweets, ensuring balanced representation across categories. EDA creates new data by substituting words with synonyms, randomly deleting words, and swapping word positions.

Using the training, set we trained a separate model for each category and calculated the F1-score on the test set. The models achieved the following F1-scores across categories: classifiable/non-classifiable (0.76), user (0.75), medical or non-medical (0.88), legalization (0.82), health risks (0.79), discussion of therapeutic or medicinal use (0.88), content on preventive measures (0.5), sentiment regarding consumption (0.7), and type of consumption (0.91). Due to the low predictive quality, we excluded the model for “content on preventive measures” from our analysis. However, the other categories achieved satisfactory F1-scores, indicating strong model performance.

Finally, emotion analysis was conducted using a pretrained neural network called emotion-english-distilroberta-base (34), capable of detecting six basic Elkman’s emotions (35) and neutral sentiment. This network, previously used in research studies, was applied to the preclassified tweets. This methodology has been correctly validated, already (36, 37).

### 2.5 Statistical analysis

First, a descriptive study was conducted for all collected variables. For categorical variables, absolute and relative frequencies were calculated. For quantitative variables, the normality of the distribution was initially assessed using the Kolmogorov–Smirnov test and graphical representations. Since the variables did not follow a normal distribution, the median and interquartile range were used to summarize the results. Second, bivariate analyses were performed to answer the research questions. For the cross-tabulation of categorical variables, the Pearson’s chi-squared test or Fisher’s exact test was used, as appropriate. In cases where a quantitative variable was compared with a categorical variable, the Mann–Whitney U test (for bivariate comparisons) or the Kruskal–Wallis test (for three or more categories) was employed. No *p*-value adjustment was performed, with a significance level of <0.05 considered statistically significant. The obtained *p*-values were reported in all cases. The analysis of results was conducted using IBM SPSS Statistics version 27.

**Figure 1:**
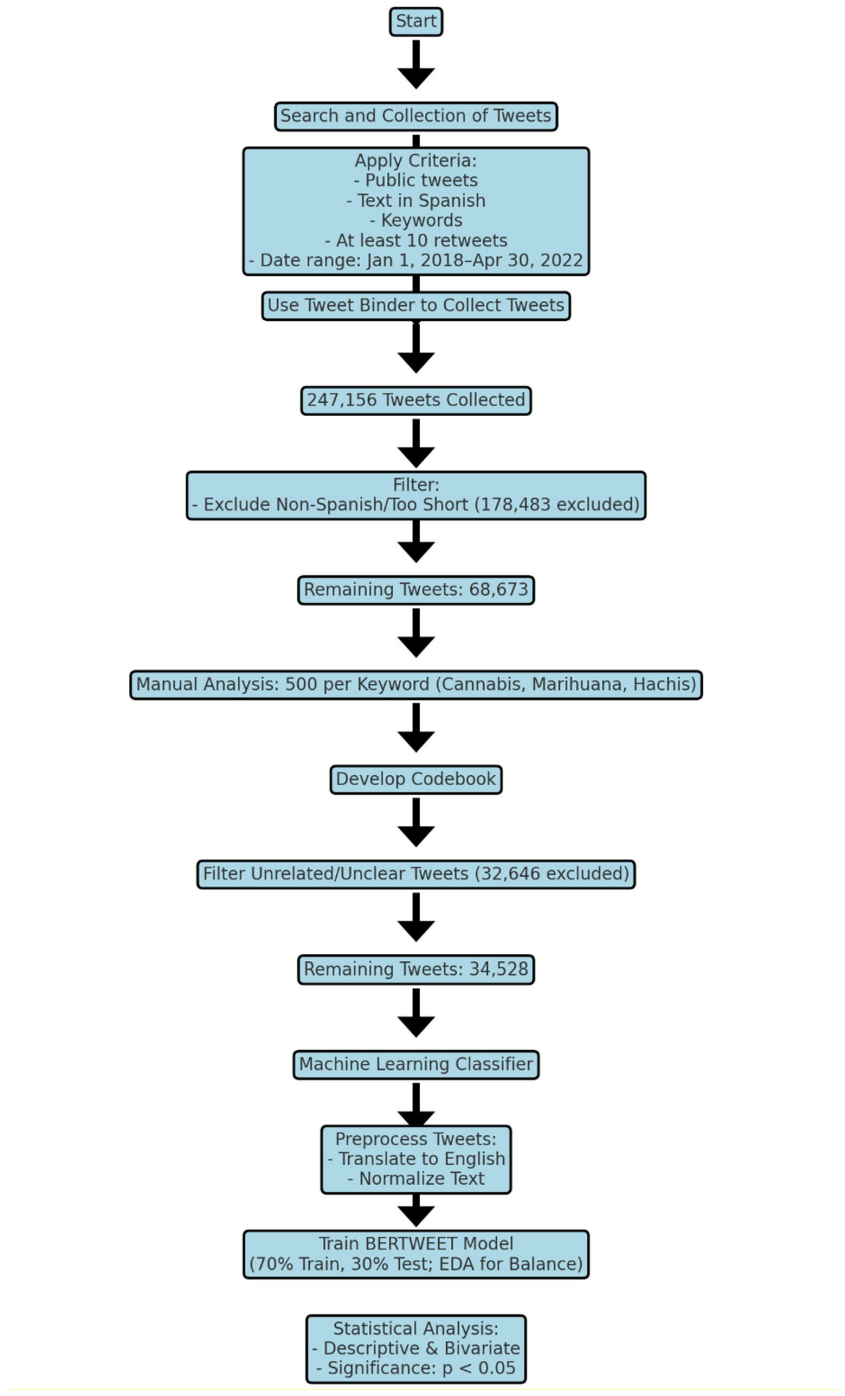
Flow chart of the methodology followed in this work.

Overall in Figure 1 we provide a flow chart of the methodology used in this study.

## 3 Results

### 3.1 General description of analyzed tweets

Table 2 provide a global description of the analyzed tweets. Firstly, we observed that tweets containing medical information represented a 47.1% of the total tweets whereas those that do not include medical information represented the remaining 52.9%. In parallel, our study reports that most tweets came from general users (anonymous counts) of Twitter (61.6%), followed by media (20.3%), although up to a 10.6% of the tweets were not identified.

**Table 2:** Distribution of tweets according to the shared content.

Topic	Frequency *N* (%)
Classificable	
Medical information	16,246 (47.1%)
Non-medical information	18,282 (52.9%)
Total	34,528 (100%)
User type	
Indeterminate	3,673 (10.6%)
General user	21,275 (61.6%)
Media	7,022 (20.3%)
Public figures	2,558 (7.4%)
Total	34,528 (100%)
Risks and harm to physical and mental health	
No	27,478 (79.6%)
Yes	7,050 (20.4%)
Total	34,528 (100%)
Legalization	
Neutral	8,038 (23.3%)
In favor	25,290 (73.2%)
Against	1,200 (3.5%)
Total	34,528 (100%)
Therapeutic use	
Against	24,122 (69.9%)
In favor	10,406 (30.1%)
Total	34,528 (100%)
Emotions regarding consumption	
Negative or neutral	28,563 (82.7%)
Positive	5,965 (17.3%)
Total	34,528 (100%)
Type of consumption	
Non consumer or unknown	33,650 (97.5%)
Consumer	878 (2.5%)
Total	34,528 (100%)

**Figure 2:**
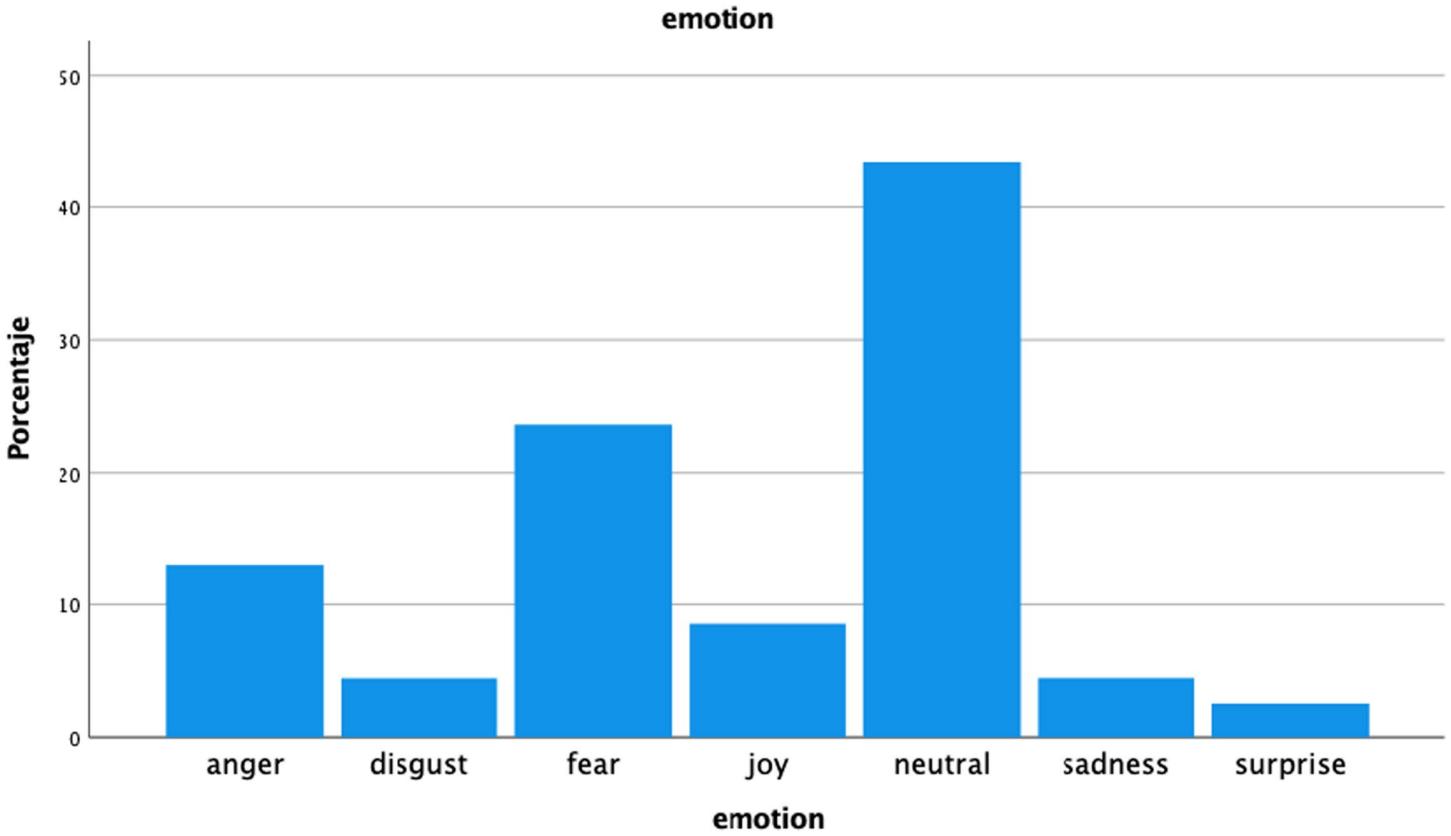
Distribution of tweets according to the expressed emotion around cannabis content.

We also observed that 17.3% of tweets express positive emotions around cannabis, whereas the remaining 82.7% included neutral or negative emotions. Excluding neutral opinions, the most frequently observed emotion was fear (Figure 2). Finally, only 2.5% openly discussed personal cannabis consumption, while 97.5% do not.

### 3.2 A significant percentage of tweets express support for the legalization of cannabis, overlooking both the potential harms of this substance and its therapeutic use

Regarding legalization, we observed that 73.2% of the analyzed tweets were in favor and only a 3.5% of the population expressed against its regulation (Table 2). The remaining 23.3% of tweets were neutral around this question. In terms of health-related aspects of cannabis, we observed that only 20.4% of the tweets discussed negative effects of cannabis for physical or mental health. The remaining 79.6% do not discuss or mention any danger associated with its consumption. In paralell, 30.1% of the tweets are in favor of the therapeutic use of cannabis, while 69.9% of tweets manifests neutral or against therapeutic use (Table 2).

When we deepen on legalization and health issues related to cannabis consumption, our study reveals that the most active users on the platform are those advocating for the legalization and unrestricted consumption of cannabis (Table 3). Tweets originating from self-proclaimed cannabis consumers demonstrated the highest reach, as evidenced by a substantial number of retweets and likes. The predominant content in these tweets was characterized by strong support for cannabis legalization, along with the promotion of purported benefits associated with its use.

**Table 3:** Distribution of tweet categories based on the number of retweets and likes received.

Topic	Retweets	Likes	*p* value
Risks and harm to physical and mental health			
No	17,148.50	17,223.16	<0.001
Yes	17,711.68	17,391.43	
Legalization			
Neutral or against	12,938.27	12,972.81	<0.001
In favor	19,697.88	18,912.68	
Therapeutic use			
Neutral or against	17,858.16	24,115	<0.001
In favor	15,884.96	10,399	
Emotions regarding consumption			
Negative or neutral	17,115.22	17,097.53	<0.001
Positive	17,973.49	18,023.89	
Type of consumption			
Non consumer or unknown	17,188.49	17,074.35	<0.001
Consumer	20,138.17	24,273.93	
Classificable			
Medical information	16,555.39	16,184.68	<0.001
Nonmedical information	17,892.74	18,210.35	
Type of user			
General user	15,430.87	15,999.24	<0.001
Media	14,322.82	12,940.35	
Public figures	18,425.50	17,419.78	

The majority of individuals opposed to cannabis legalization firmly believe that it should not be considered as a therapeutic option (Table 4). Conversely, among those in favor of legalization, only 34.6% believe in its potential bodily benefits, while 65.5% do not, suggesting a prevalent recreational intent. Additionally, both proponents and opponents of legalization exhibit skepticism regarding the potential adverse effects on physical and mental health, with 93.8 and 95%, respectively, expressing disbelief in such risks.

**Table 4:** Distribution of tweets regarding the therapeutic use of cannabis and the health risks associated with its consumption in subjects with neutral views, in favor and against cannabis legalization.

Legalization	Neutral	In favor	Against	*p*-value
Therapeutic use				
Against	6,386 (79.4%)	16,548 (65.4%)	1,188 (99%)	<0.001
In favor	1,652 (20.6%)	8,742 (34.6%)	12 (1%)	
Risks/harms for physical and mental health				
No	2,628 (32.7%)	23,710 (93.8%)	1,140 (95%)	<0.001
Yes	5,410 (67.3%)	1,580 (6.2%)	60 (3%)	

In general, X users are against therapeutic use of cannabis, specially in those against its legalization, whereas almost two third of users in favor of cannabis legalization are also against its therapeutic use. Regarding the perceived risk/harms for physical and mental health, a significant number of subjects identified as neutral in terms of cannabis legalization believe that cannabis is detrimental for health, whereas both proponents and opponents of legalization express disbeliefs in such risks.

### 3.3 A comparative analysis reveals diverse backing for legalization, varied stances on health risks and medicinal use according to the user type

As shown in Table 5, from the three identified user types, over 75% do not reference the risks associated with cannabis or firmly believe that such harm to health does not exist. Regarding legalization, we observe that in all three user types, the highest percentages (74.3, 73.3, and 60.9% respectively) indicate that they are in favor of cannabis being regulated by the state.

**Table 5:** Distribution of tweets according to the type of user.

Topic	General user *N* (%)	Media *N* (%)	Public figures *N* (%)	*p*-value
Risks and harm to physical and mental health				
No	16,678 (78.4%)	5,275 (75.1%)	2,410 (94.2%)	<0.001
Yes	4,597 (21.6%)	1,747 (24.9%)	148 (5.8%)	
Legalization				
Neutral	5,344 (25.1%)	1,850 (26.3%)	84 (3.3%)	<0.001
In favor	15,889 (74.3%)	5,169 (73.3%)	1,557 (60.9%)	
Against	42 (0.2%)	3 (0%)	917 (35.8%)	
Therapeutic use				
Neutral or against	14,612(68.7%)	4,441 (63.2%)	2,332 (90.8%)	<0.001
In favor	6,663 (31.3%)	2,581 (36.8%)	236 (9.2%)	
Emotions regarding consumption				
Negative or neutral	17,285 (81.2%)	5,600 (79.7%)	2,473 (96.7%)	<0.001
Positive	3,990 (18.8%)	1,422 (20.3%)	85 (3.3%)	
Consumers				
No or unknown	20,527 (96.5%)	7,010 (99.8%)	2,556 (99.9%)	<0.001
Yes	748 (3.5%)	12 (0.2%)	2 (0.1%)	

Regarding medicinal cannabis, all three user types (68.7, 63.2, and 90.8%) are against the use of cannabis for therapeutic purposes. This suggests that, considering the previous observation that the majority support its legalization, it is likely for recreational purposes.

All three user types have a neutral or negative sentiment toward cannabis consumption, with over 79.9% expressing such sentiment.

### 3.4 Differing perspectives on risks, legalization, medicinal use, and sentiments among consumers and non-consumers

**Figure 3:**
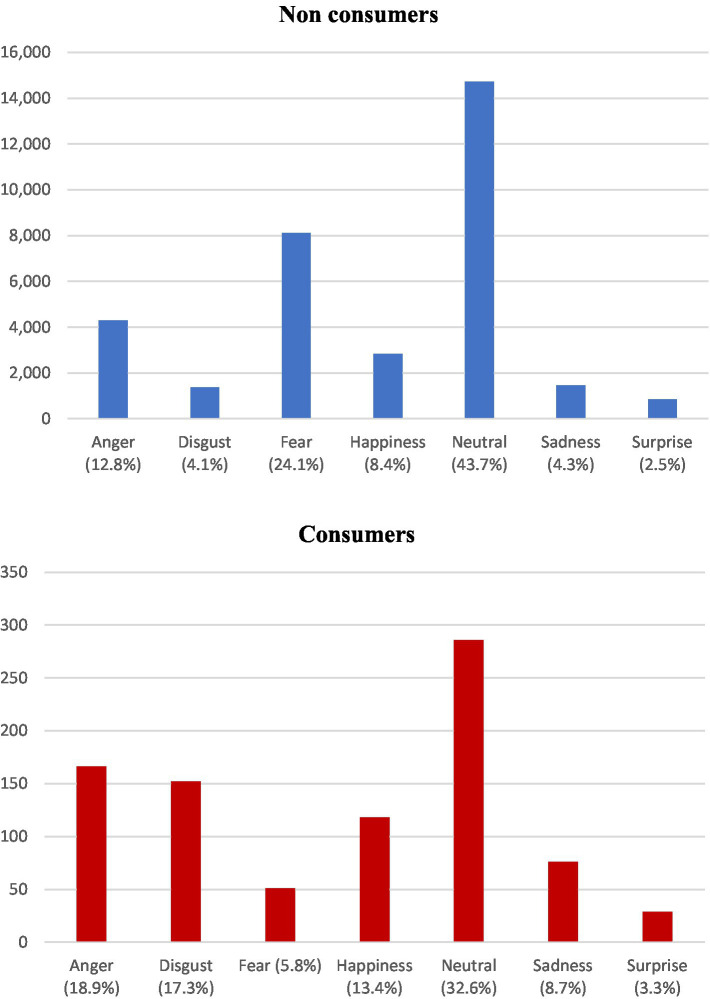
Emotion analysis in non-consumers (blue) versus consumers (red).

Table 6 shows a comparative between consumers and non-consumers. It is noticeable that non consumers are less likely to discuss risks and harms of cannabis consumption in comparison to consumers (20.2% versus 27.7%) whereas non-consumers advocate for cannabis legalization more frequently than consumers do (75% versus 4.7%). In both groups, they believe that cannabis could be used as a medical alternative to treat various conditions. It is worth noting that this is much more treated in non-consumers (30.8% versus 4%). Regarding the emotions toward consumption, almost 84% of non-consumers manifest negative or neutral views, with only 16.3% expressing positive feelings in comparison to up to a 55.2% of consumers. It is of note that non consumers are more likely to exhibit neutral emotions that consumers (43.7% versus 32.6%, Figure 3). When distinguishing the analyzed emotions we observed that appart from neutral sentiments, non-consumers more commonly manifest fear (24.1%), anger (12.8%) and happiness (8.4%), whereas consumers more commonly exhibit anger (18.9%), disgust (17.3%) and happiness (13.4%) (Figure 3).

**Table 6:** Distribution of tweets classified according to the type of consumer.

Topic	Non-consumer	Consumer	*p*-value
Risks and harm to physical and mental health			
No	26,843 (79.8%)	635 (72.3%)	<0.001
Yes	6,807 (20.2%)	243 (27.7%)	
Legalization			
Neutral	7,201 (21.4%)	837 (95.3%)	<0.001
In favor	25,249 (75%)	41 (4.7%)	
Against	1,200 (3.6%)	0 (0%)	
Therapeutic use			
Neutral or against	23,279 (69.2%)	843 (96%)	<0.001
In favor	10,371 (30.8%)	35 (4%)	
Emotions regarding consumption			
Negative or neutral	28,170 (83.7%)	393 (44.8%)	<0.001
Positive	5,480 (16.3%)	485 (55.2%)	

### 3.5 Geolocalization data reveals that most tweets written in Spanish discussing cannabis came from Spain, Chile, Argentina, Mexico and Colombia

**Figure 4:**
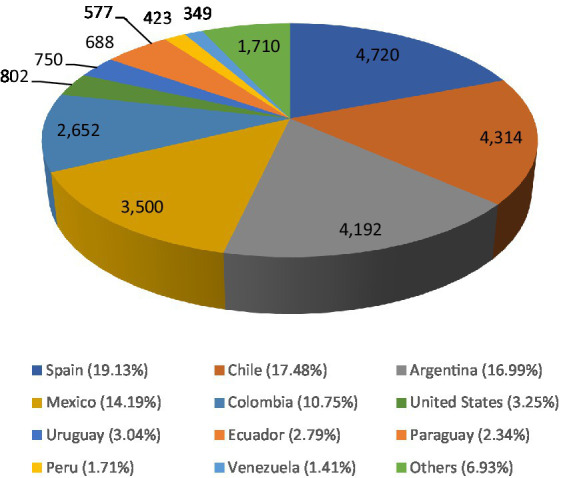
Distribution of tweets by country.

Out of the total analyzed tweets, 73.3% had geolocation data available. Of the localized tweets, 78.54% originated from five countries: Spain (19.13%), Chile (17.48%), Argentina (16.99%), Mexico (14.19%), and Colombia (10.75%). Other regions geolocalized in our study was United States, Uruguay, Ecuador, Paraguay, Peru and Venezuela (Figure 4).

## 4 Discussion

In this study, we have observed that Twitter users show a great interest in cannabis, focusing mainly on regulatory aspects of the substance as well as health-related aspects. The most active users are those who are in favor of its legalization and unrestricted consumption. Tweets written by users who claimed to consume cannabis have had the highest reach, meaning they received a greater number of retweets and likes, and their content mainly supported the legalization and promoted the alleged benefits of cannabis. On the other hand, healthcare professionals did not have a strong presence in driving Twitter conversations regarding cannabis, nor did medical institutions or government organizations.

Firstly, we observed that 73.2% of the posts we analyzed are in favor of cannabis legalization. According to previous works, the main reasons explaining the public support of cannabis legalization was to state that this drug is less dangerous than other substances and has significant medical benefits, also considering criminal justice reform and the potential for tax revenue as potential benefits of legalization (38). Besides, in the same study the authors also found that harms associated with cannabis use were the most commonly reasons for opposing legalization. Likewise legalizing cannabis could contribute to a lower perception of risks and reduce the fear in the population, potentially encouraging increased consumption (39). Indeed, available literature reports that this has occurred in certain regions like the United States, where there appears to be a ~ 20% average increase in cannabis use frequency attributable to recreational legalization (40). According to the NCBI, cannabis consumption has increased among adults over 21 years of age in countries like Canada and the United States that have passed laws allowing its medical use, leading to higher rates of daily use, misuse, and dependence (41, 42). Additionally, the number of adult men seeking treatment for cannabis-related disorders has increased more in these countries. Howsoerver, compelling evidence supports that public engagement with information about medical cannabis in the internet and social media are one of the main mechanisms by which medical cannabis legalization is associated with cannabis legalization (43). In this sense, our study seems to be in line with these claims, and Twitter could be a platform reflecting the complex social environment surrounding cannabis legalization and medical use.

It was also surprising for us that up to 79.6% of tweets do not perceive the risk of consuming cannabis, stating that it is not associated with any harmful health risks. The perception of cannabis as harmless often stems from its “natural” origin; however, this does not imply physiological safety, as cannabis exerts significant psychoactive effects, primarily from THC, which acts quickly on the brain (44, 45). This is particularly concerning among youth, especially those with coexisting mental or substance abuse disorders (46–49). Cannabis use is linked with a range of acute and chronic adverse effects, including hyperemesis syndrome, anxiety, and long-term neurocognitive, cardiovascular, and respiratory issues, with risks escalating with earlier and more frequent use (50, 51). Importantly, the connection between cannabis use and psychosis is well-documented but often underrepresented in both public discourse and policy. Evidence shows that high-potency cannabis use significantly increases the risk of developing psychotic disorders, especially in vulnerable populations. For instance, the EU-GEI multicenter case–control study found that differences in cannabis use patterns across European cities were significantly associated with variations in the incidence of psychotic disorders (7). Furthermore, early cannabis use has been shown to act as a modifiable environmental risk factor for psychosis onset, alongside genetic and social variables (52) Moreover, cannabis use in individuals with early psychosis is associated with increased risk of relapse and hospitalization, although recent studies indicate that treatment with long-acting injectable antipsychotics, such as aripiprazole, may help reduce these risks and improve quality of life (53, 54).

Likewise, cannabis consumption over time can lead to a situation of dependency named cannabis use disorders (CUD) (55, 56). CUD commonly co-occurs with other mental health disorders, increasing risks of self-harm, overdose, and mortality among youth with mood disorders (41). The risk of developing this type of addiction seems to be greater in individuals aged between 13 to 18 years old (57). Additionally, cannabis use disorder is a common comorbidity and a risk marker for self-harm, all-cause mortality, unintentional overdose, and homicide among young people with mood disorders (58). While no specific treatment for CUD exists, symptom-targeted medication, psychotherapy, and psychoeducation are recommended, especially for adolescents. Furthermore, the use of unregulated or unpurified cannabis extracts presents additional health concerns. These products often lack standardized labeling and may inaccurately report the concentrations of key cannabinoids such as THC, CBD, (59, 60). This can mislead consumers about the potency and potential effects of the extract. In some cases, unregulated extracts may contain other bioactive compounds like terpenes, flavonoids, or alkaloids, which can alter the pharmacological profile of the product, potentially enhancing psychoactive effects or increasing the risk of adverse reactions. Without appropriate oversight and quality control, such formulations pose unpredictable health risks, particularly when consumed by vulnerable populations (60). Addressing the low-risk perception and social allure of cannabis use is key to improving health outcomes, as smoked cannabis poses notable acute and chronic health risks (8).

On the other hand, the medicinal use and health benefits of cannabis were also considered in an important percentage of tweets. Surprisingly, our results show that despite most tweets being in favor of cannabis legalization and perceiving a low risk of its use, almost 70% of tweets did not consider or rejected the therapeutic use of cannabis. It is important to highlight that a growing number of studies have shown multiple benefits from cannabinoids, especially the compound named cannabidiol (CBD), another component found in the *Cannabis sativa* plant. Initial evidence supports its effects to alleviate insomnia, inflammation, anxiety, depressive symptoms, pain, post-traumatic stress disorder, and so on (61–63), especially in form of CBD and hemp oils. However, the existing literature claims that there is little regulation around these products and studies have found inaccurate labeling of CBD and THC quantities (64). Besides, further clinical research is required, as well as to find adequate doses and applications for each subject and explored condition (65). Currently, the FDA has approved the use of synthetic cannabis-related drug products, mainly two compounds containing dronabinol (a synthetic form of THC) for the treatment of anorexia associated with weight loss in AIDS patients, another product with nabilone (THC) prescribed for the treatment of nausea and vomiting associated with anticancer chemotherapy and the proper CBD, which can be used to manage and treat the seizure disorders Lennox–Gastaut syndrome and Dravet syndrome (66). Also, there is moderate evidence from the use of cannabis and cannabinoids for pain relief in patients with chronic pain and for treating multiple sclerosis (MS)-related spasticity (67). Overall, medical uses and benefits from cannabis, especially in the form of CBD are increasingly being supported, although it is also true that many of these properties have been overclaimed and further regulation is required before widespread use of these components, evidencing the need of contextualizing this complex picture in social media like Twitter.

In our study, only 2.5% openly discuss their consumption, whereas the remaining 97.5% were identified as non-consumers. When compared to non-consumers, consumers were more likely to discuss detrimental physical/mental health effects of cannabis (27.7% versus 20.2%). Conversely, we observed that, unlike consumers, non-consumers tended to show in favor of cannabis legalization (75% versus 4.7%) and its therapeutic use (30.8% versus 4.8%), also exhibiting more commonly negative feelings around cannabis consumption (83.7% versus 44.8%). Previous works conducted on Twitter found an important number of tweets discussing unsustained health benefits derived from cannabis use, especially those tweeted by social bots (17). The authors reported that this type of information might be influencing Twitter users to perceive CBD and cannabis as anticancer and effective treatments for several diseases, whereas the level of evidence regarding their uses remains to be fully investigated in most cases. Thus, it would be reasonable that these types of beliefs and ideas could influence the legal debate around cannabis use. However, it was surprising for us that, of those subjects in favor to legalize cannabis consumption, only 35% knew and supported the therapeutic use of cannabis, suggesting that most tweets in favor of cannabis legalization were related to its recreational use. Also, the fact that an important percentage of non-consumers support cannabis legalization could also indicate that an important part of Twitter users are not knowledgeable about the acute and chronic effects of recreational cannabis consumption, as well as the number of individuals affected by such use and the complex relationships that exist between legalization and the social perception of a drug. The therapeutic effects of cannabinoids are exerted mainly through the modulation of cannabinoid receptors (CB1 and CB2) and the endocannabinoid system (ECS). The ECS system has a negative feedback mechanism and retrograde signaling to maintain physiological balance. Exogenous cannabinoids (like THC and CBD) can enhance or prolong ECS effects, also activating other receptors beyond CB1 and CB2, including transient receptor potential (TRP) channels, such as TRPV1, peroxisome proliferator-activated receptors (PPARs), or other non-cannabinoid targets like GPR55, GPR18, and serotonin receptors, all of which contribute to the broad pharmacological profile of cannabinoids in various therapeutic contexts, including epilepsy, multiple sclerosis, anxiety, and chronic pain (68–71). Understanding these diverse molecular pathways is key to evaluating both the potential medical benefits and associated risks of cannabinoid-based treatments. Because of this, there are authors that claim the need for designating timely social media communications with new cannabis-related information by authoritative institutions that deal with public health in order to help a general public mostly exposed to pro-cannabis content on Twitter (72).

Finally, when we considered geolocalization data, most of the tweets (56.1%) were from Spain and Hispanic American countries, with Chile, Argentina, Mexico, and Colombia representing up to 42.4% of the analyzed tweets. Without considering neutral opinions, our sentiment analysis showed that fear and anger were the most common manifestations expressed on Twitter. Previous works conducted in Twitter have evidenced the relevance from considering geographic differences in the sentiment and content of cannabis-related tweets, specially due to the different legislation and/or reported consumption in different regions (73). On the one hand, in Spain, the medical use of cannabis is already legal, but it is regulated and has nothing to do with recreational use (74). However, as previously mentioned, Spain is ranked as the third European country in cannabis consumption after France and Denmark, and the recreational uses of this drug is a growing concern affecting this country, especially among youth and men (75). Thus, Twitter may clearly reflect the associated feelings of worry with this situation. On the other hand, it seems that there is a huge heterogeneity in attitudes toward drug policies in Hispanic American countries. According to the 2014 Annual Survey of the Observatory of Drug Policies and Public Opinion (76), a notable diversity in perspectives pertaining to drug policies is observable across South America countries, indicative of a discernible scrutiny directed toward the prevailing norms in the region. In the case of Mexico, Argentina, Colombia and Chile, an important percentage of surveyed people are in favor of recreational uses of cannabis, whereas Chile, Colombia and Mexico ranked as the first, second and fourth Latin American countries with more people supporting cannabis legalization, with more of the 40% of the people surveyed Despite only 26.4% of people in Argentina were in favor of cannabis legalization, this country obtained the second-highest rate of people who had ever consumed cannabis after Chile (76). To this complex picture, it should also be considered the regulatory framework around medical use of cannabis, as it is also a matter of concern in these regions (77) and the fact that some of these countries like Chile and Colombia are among those with the highest incidence of cannabis use disorder in Latin American (78). Overall, our results agree with the complex background around cannabis consumption, legalization and recreational issues observed in these countries, explaining why the fear and anger are the predominant feelings in our observed tweets.

## 5 Limitations

This study has some limitations. Firstly, since Twitter users tend to be younger than the general population, it’s possible that our results may not apply to older age ranges. In fact, adolescent patients, although experiencing an exponential growth of psychotic outbreaks and hospital admissions for this reason, develop other diseases such as cancer or COPD at a later age. Secondly, we were unable to examine how clinical characteristics, symptom severity, duration of use, or residual cognitive dysfunction associated with marijuana influenced the content of the social media posts due to a lack of psychiatric evaluation. Thirdly, the coding book and text analysis we used involve a degree of subjectivity. However, this methodology is consistent with previous medical research studies on Twitter and could be applied to various topics by different authors. Fourthly, the list of keywords included generic terms, but tweets that contained spelling errors may have been excluded. Lastly, incorporating studies that analyze cannabis content on platforms like Facebook, Instagram or TikTok could enhance the accuracy of our understanding of public perceptions of these drugs on social media and within the broader population (79–81).

## 6 Conclusion

Our findings highlight the potential of leveraging social media to better understand the rise of cannabis as a drug of choice in our population. As a preventive measure in a society that is increasingly in favor of approving a law for the free consumption of cannabis, it is important for healthcare professionals and medical and political authorities to intervene by publishing more content about the risks of its consumption and doing so more frequently. These findings should be considered as states consider the legalization of medical and recreational marijuana, as both are associated with increased cannabis consumption and related risks. An increased risk of cannabis use disorder. Twitter can also serve as an additional educational tool to raise awareness among its users about the current status of therapeutic and medical uses of cannabis and, above all, its numerous disadvantages and consumption risks primarily derived from recreational and inhalation use.

## Data Availability

The raw data supporting the conclusions of this article will be made available by the authors, without undue reservation.
